# A Mapping Review of the Pathogenesis of Peri-Implantitis: The Biofilm-Mediated Inflammation and Bone Dysregulation (BIND) Hypothesis

**DOI:** 10.3390/cells13040315

**Published:** 2024-02-08

**Authors:** Ethan Ng, John Rong Hao Tay, Nikos Mattheos, Nagihan Bostanci, Georgios N. Belibasakis, Chaminda Jayampath Seneviratne

**Affiliations:** 1 Department of Restorative Dentistry, National Dental Centre Singapore, Singapore 168938, Singapore; john.tay.r.h@singhealth.com.sg; 2 Department of Oral and Maxillofacial Surgery, Faculty of Dentistry, Chulalongkorn University, Bangkok 10330, Thailand; nikos@mattheos.net; 3 Division of Oral Health and Periodontology, Department of Dental Medicine, Karolinska Institute, 14152 Stockholm, Sweden; nagihan.bostanci@ki.se (N.B.); george.belibasakis@ki.se (G.N.B.); 4 School of Dentistry, The University of Queensland, Brisbane, QLD 4006, Australia; 5 School of Dentistry, Center for Oral-Facial Regeneration, Rehabilitation and Reconstruction (COR3), The University of Queensland, Brisbane, QLD 4072, Australia; 6 National Dental Research Institute Singapore, National Dental Centre Singapore, Singapore 168938, Singapore

**Keywords:** peri-implant disease, osseointegration, dysbiosis, biofilms, bone remodelling

## Abstract

This mapping review highlights the need for a new paradigm in the understanding of peri-implantitis pathogenesis. The biofilm-mediated inflammation and bone dysregulation (BIND) hypothesis is proposed, focusing on the relationship between biofilm, inflammation, and bone biology. The close interactions between immune and bone cells are discussed, with multiple stable states likely existing between clinically observable definitions of peri-implant health and peri-implantitis. The framework presented aims to explain the transition from health to disease as a staged and incremental process, where multiple factors contribute to distinct steps towards a tipping point where disease is manifested clinically. These steps might be reached in different ways in different patients and may constitute highly individualised paths. Notably, factors affecting the underlying biology are identified in the pathogenesis of peri-implantitis, highlighting that disruptions to the host–microbe homeostasis at the implant–mucosa interface may not be the sole factor. An improved understanding of disease pathogenesis will allow for intervention on multiple levels and a personalised treatment approach. Further research areas are identified, such as the use of novel biomarkers to detect changes in macrophage polarisation and activation status, and bone turnover.

## 1. Introduction

Since the discovery of osseointegration, the field of implant dentistry continues to advance semper prorsum—from implant material modifications to improve osseointegration, to treatment digitalisation such as impression making using scan bodies [1] and dynamic and static computer-assisted implant surgery [2]. With appropriate patient selection and follow-up, dental implants are a predictable replacement option for missing teeth with positive long-term clinical performance being reported for as long as 20 years in the literature [3,4,5]. Nevertheless, dental implants are also susceptible to the same inflammatory processes and risk factors that may have contributed to the patient’s initial tooth loss. Biological complications in the form of peri-implant mucositis or peri-implantitis are not uncommon occurrences [6,7]. Collectively termed as ‘peri-implant diseases’, these conditions are characterised by the inflammatory destruction of implant-supporting tissues and are induced by the implant-associated microbial biofilm [8].

As peri-implant mucositis is considered to precede peri-implantitis in a similar way to gingivitis and periodontitis, much of the research on the aetiology of peri-implant mucositis has focused mainly on biofilm control and the shared similarities in risk indicators [9,10]. However, there is scarce evidence on how peri-implant mucositis transitions to peri-implantitis, and the features or conditions resulting in conversion from peri-implant mucositis to peri-implantitis are not completely understood [11]. In peri-implantitis inflammatory lesions, the microbial composition and qualitative composition of immune cells resemble established periodontitis lesions [12,13]. Peri-implant soft tissues also develop a stronger inflammatory response to microbial biofilms [14,15], with disease progressing in a non-linear and accelerating pattern [16]. While these observations could be due to structural differences in the surrounding tissues of a tooth and an implant, they add little to the understanding of its pathogenesis or conversion to a diseased state.

Immune response to bacterial dysbiosis could vary between individuals due to different risk factors that may influence bone biology and inflammation. Indeed, the literature describes the possibility of cluster patterns in implant failure, where a minority of patients account for most of the implant failures in a cohort [17,18]. This phenomenon suggests that there might be patient-specific and even implant design-specific features that may be predictors of implant failure [19].

Periodontitis is a polymicrobial disease associated with global community dysbiosis related to shifts in community structure, rather than shifts in bacteria membership [20]. It is associated with the pathologic loss of the tooth attachment apparatus and should not be considered as a bacterial infection, but an eco-genetic disease where genetic and environmental risk factors play a complex role that leads to chronic hyperinflammation at the histopathological level, prior to the presentation of clinical disease [21,22]. Similarly, when considering the pathogenesis of peri-implantitis, factors that disrupt bone homeostasis and upregulate inflammatory pathways should be primarily considered. This review primarily aims to map a framework for peri-implantitis pathogenesis, explaining how different potential factors may form multiple stable states preceding a ‘tipping point’ in clinical forms of peri-implant disease. The secondary aims are to discuss a potential biologic ‘switch’ that drives the disease process and the clinical implications of the presented framework.

## 2. The Biofilm-Mediated Inflammation and Bone Dysregulation (BIND) Hypothesis

**Figure 1:**
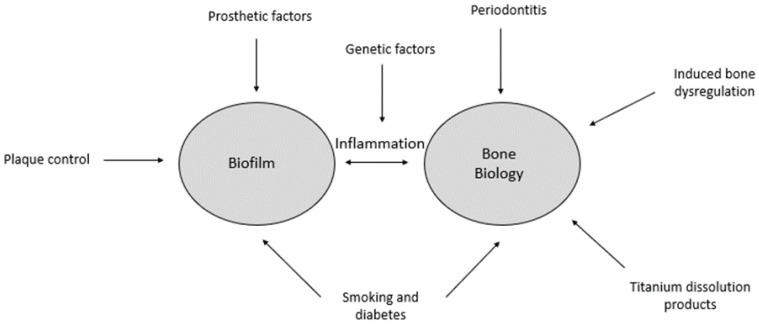
The BIND hypothesis. Bone biology and biofilm are influenced by several possible factors. These two core components are linked by the inflammatory process, which may be modified by genetic factors. Microbial challenges from biofilms, exacerbated by factors such as poor plaque control and prosthetic design, may disrupt the physiologic equilibrium by upregulating inflammatory mediators to induce osteolytic activity. On the other hand, disturbances to bone biology that induce bone dysregulation could have a synergistic effect with inflammation-induced plaque accumulation, thus exacerbating chronic inflammation at the site. Bone dysregulation is defined here at a molecular level and may not necessarily translate to clinical signs of peri-implant disease unless it is tipped to a clinical state of disease through the process of hysteresis.

The first part of the BIND hypothesis maps the aetiology of peri-implantitis and proposes that interactions between microbial dysbiosis, immune cells, and bone cells around the implant drive the pathological process (Figure 1). The second part provides a framework for clinical disease progression based on the concept of a ‘multi-stable system’ or ‘multistability’ (see Table 1 for a description of the terms).

‘Multi-stability’ implies more than one stable state under the same conditions, and this has been suggested as a mechanism behind different microbial community types despite similar environmental conditions [23]. While this concept has usually been described in microbial models [24,25,26], multiple stable states in the oral microbiota have recently been described in a human cohort [27]. In a multi-stable state system, each state is associated with a different species abundance profile and is established by competition for nutrients and mutual complementarity [24]. These stable states are generally resilient to changes, but perturbations beyond a threshold level may result in a transition to another state and cannot be regained by simply reversing the initial perturbation. The innate resistance in a system before transitioning to a different state has been described as hysteresis using the analogy of a frictionless seesaw and seesaw with friction [26], and this is a telltale sign of multi-stability [23]. Briefly, a frictionless seesaw is a multi-stable system with two states determined by the position of the person. On the other hand, a seesaw with friction is a multi-stable system with hysteresis, where the state of the system does not change while the position of the person remains in the region of hysteresis.

**Figure 2:**
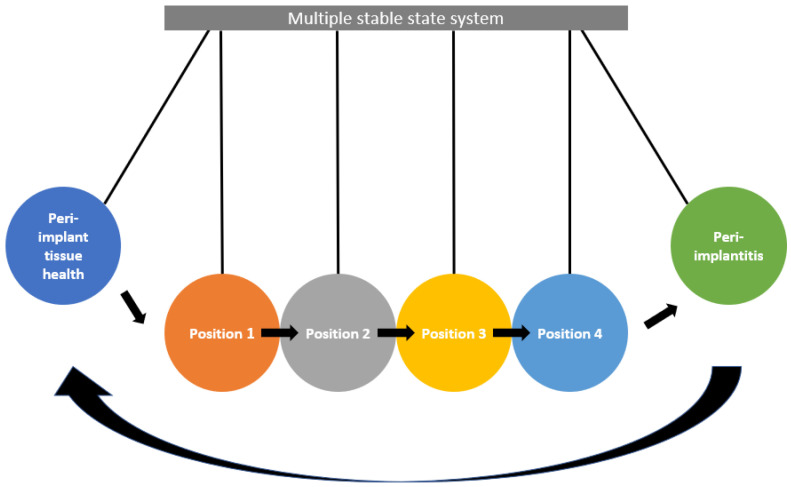
The multi-stable state system. Different perturbations place implant sites at different positions from the tipping point toward disease. However, deviations from peri-implant tissue health may not present as disease, resulting in multiple stable states of clinical health. For example, position 1 could represent a non-susceptible individual with few factors in the bone biology category and some factors in the biofilm category, and never display clinical signs of peri-implant disease. Position 2 could be a non-susceptible individual with more factors in either category but still never have clinical signs of peri-implant disease. Position 3 could represent a susceptible individual with some factors in the bone biology category but few risk factors in the biofilm category. These individuals may develop early disease (mucositis), and possibly progress to clinical signs of peri-implantitis in the future. Position 4 is a susceptible individual with more factors in both the bone biology and biofilm category, and thus would be most likely to develop peri-implantitis. These states are dynamic and can change within individuals. The reverse arrow suggests that it is possible to return from a disease state with intervention, and the endpoint may be any of the positions in the multiple stable state system.

Applying this multi-stable state system to peri-implantitis, different perturbations could place individual implant sites at different ‘stable’ positions before the tipping point towards health or disease (Figure 2). For example, multiple positions may be speculatively determined by non-susceptible or susceptible individuals with varying levels of risk factors involving early bone loss or biofilm control, resulting in a profile closer to developing peri-implantitis. In support of this model, a linear microbial succession was observed from healthy to clinically diseased implants [28]. Peri-implant health requires the absence of clinical signs of inflammation and the absence of bleeding or suppuration on gentle probing [29]. Consistent with the prevailing understanding that mucositis precedes peri-implantitis, the proposed model is also linear. It is likely that mucositis and successfully treated peri-implantitis could exist within the ‘hysteresis’ states, especially if a flexible case definition allows for the mild inflammation of ≤2 dots of light bleeding on probing [30] or ≤1 point of bleeding or probing [31] are considered as endpoints of treatment. A discussion of the various factors influencing this multi-stable state system is described below.

## 3. Bone Biology

Bone is a mineralised connective tissue consisting of four different cell types—osteoblasts, osteocytes, osteoclasts, and bone-lining cells [32]. Cellular communication between osteoblasts and osteoclasts, in particular, is essential for bone homeostasis to maintain bone integrity and is governed by two fundamental processes [33]. The first process is bone remodelling, where the existing bone is resorbed by osteoclasts and replaced in the same location by new bone laid down by osteoblast; and the second is bone modelling, where bone formation and resorption occur at different sites [34]. Bone modelling occurs during growth or in response to mechanical loading and may be influenced by pharmacological agents [35]. Osteocytes are terminally differentiated osteoblasts embedded in bone matrix and constitute 90–95% of all bone cells [36]. Their functions include functional adaptation of bone in response to mechanical strain, and the regulation of bone turnover [37]. At the implant–bone interface, osteocytes are frequently identified histologically, sometimes in direct proximity to the metal surface [38]. Osteoblasts receive signals from osteocytes to induce bone remodelling; they exhibit an osteogenic phenotype which lays down bone matrix at a bone resorption site, and an osteoclastogenic phenotype which supports osteoclast differentiation [39]. A coupling mechanism exists between osteoblasts and osteoclasts, beginning when osteoblasts sense osteocyte cell death which results in a change of function to osteoclastogenesis. Normally, after the old bone is resorbed, osteoclasts undergo apoptosis, and the resorption cavity is filled with new bone.

Peri-implant bone formation occurs through contact and distance osteogenesis. In contact osteogenesis, osteoprogenitor cells colonise the implant surface to form osteoblasts and lay down new bone. The ingrowth of bone from lateral walls of the osteotomy is referred to as distance osteogenesis. Distance osteogenesis seems to play a larger role in the newly formed bone–implant contact, whereas contact osteogenesis appears to be influenced by triggering factors produced during distance osteogenesis, specifically BMP-2 [40]. When titanium is exposed to air, a titanium oxide layer resembling a ceramic material forms over the implant surface, sealing the surrounding tissues off from the release of titanium particles, rendering corrosion resistance, and also facilitating the adsorption of calcium and phosphate ions [41]. The clinical success of implants is dependent on the favourable behaviour of bone at this interface zone with titanium, which also serves to dissipate stresses from the implant to the bone [41]. Bone apposition to zirconia implants is similar to titanium implants [42,43,44] and also displays high wear-resistant and corrosion-resistant properties [45]. Surface modifications to zirconia implants, such as sandblasting, may also enhance bone integration to its surface [46]. Therefore, the bone-to-implant interface presents a unique biological situation and identifying factors from a multi-stable and hysteresis perspective that influence the structure of this interface are key to understanding the pathogenesis of peri-implantitis. These include factors that affect the actual interface, and the peri-implant bone quality and remodelling process.

## 4. Bone Biology Is Disrupted by Altered Peri-Implant Bone Metabolism

### 4.1. Medical History and Medications

There is limited research investigating the biology and metabolism of bone healing around dental implants, and the implications on peri-implant marginal bone loss. However, there is biological plausibility that a medical history of high cholesterol, vitamin D deficiency, and hyperglycaemia may suggest compromised bone healing if uncontrolled [47]. In particular, the use of anti-resorptives may also result in the en bloc sequestration of successfully osseointegrated implants due to dysregulated bone remodelling and altered angiogenesis [48,49]. Peri-implantitis may be related to medication-related osteonecrosis of the jaws due to the local acidic milieu from local plaque-induced inflammation, resulting in increased concentrations of bisphosphonates [50]. This in turn may have a cytotoxic effect on the periodontium, leading to peri-implantmedication-related osteonecrosis of the jaws (PI-MRONJ) [51] or implant-related sequestration (IRS) [52]. A history of anti-resorptives’ intake has also been associated with peri-implantitis lesions that are not responsive to subgingival instrumentation, and these implants were subsequently explanted [53]. However, in the study, it was not clear whether it was the intake of anti-resorptives which resulted in peri-implantitis, or vice versa. Although there is conflicting evidence on whether the use of anti-resorptives increases the risk of peri-implantitis, the low sample size and study design (case series or retrospective cohort studies) precludes any conclusive statements. The inconclusive evidence in the literature of systemic conditions or medications in causing peri-implantitis [52] may be explained by hysteresis, that they may not be sufficient causes but may result in a closer ‘tipping’ point towards disease development by dysregulating bone metabolism.

### 4.2. Smoking and Diabetes Mellitus

Smoking has negative effects on the microenvironment of implant sites [54,55]. It promotes the early acquisition and colonisation of biofilm-forming pathogens, leading to a disease-associated peri-implant microbiome, even in clinically healthy individuals [56,57]. Metagenomic sequencing has revealed that other differences in the peri-implant microbiome in smokers include greater stability and resilience, and more coordinated microbial interactions, which have adverse effects on disease pathogenesis and responsiveness to therapy [58]. The biological effects of smoking include alterations to the microvasculature and suppression of the immune system, which inhibits the vascular response to bacterial plaque [59,60]. Smokers are twice as likely as non-smokers to experience implant failure [61] and peri-implantitis [62]. The presence of smoking should be considered a factor regardless of the levels of smoking exposure, due to a lack of literature reporting patient’s levels of smoking [62].

Likewise, diabetes mellitus is another condition that has detrimental effects on the immune system and bone metabolism [63], and microbiome composition even in a state of periodontal health [64]. Recently, a meta-analysis of observation studies reported the risk of peri-implantitis to be 50% higher in diabetes patients compared to patients with no diabetes [65]. However, diabetes mellitus is not considered a contraindication for dental implant therapy under controlled conditions [66].

### 4.3. Radiation Therapy

Radiation therapy to the head and neck has been previously reported to be an explanatory variable for inflammation associated with peri-implant mucositis [67]. In patients with a history of radiation therapy, the implant-specific radiation dose has a significant impact on peri-implant bone loss, local inflammation, and plaque, after three years [68]. Even in patients with successfully placed implants, radiotherapy for head and neck cancer was negatively associated with the resorption of marginal bone at doses of >40 Gy, although this was already significantly less than at the tumour bed [69]. In another recent study, the five-year survival of implants placed in patients receiving head and neck radiation was 75%, with marginal bone resorption and peri-implantitis as significant reasons for failure [70]. These findings highlight the impact that radiation therapy has on bone biology.

## 5. Implant Biofilm May Further Disrupt Already Disequilibrated Peri-Implant Bone

In healthy patients, different clustering patterns in the oral microbiota led to the suggestion of multi-stable states, with some being closer to the tipping point of disease [71]. Comparatively, the peri-implant microbiota is less diverse than the periodontal microbiota and presents a microbiologically distinct ecosystem [72,73,74]. The implant surface structure and abutment interface, as well as corrosion products released into the surrounding tissues, contribute to creating a unique microenvironment that drives microbial adaptation and selection [75]. Significant differences in composition are observed between healthy and diseased implants [76,77]. The peri-implantitis microbiota is a heterogeneous mixed infection that is commensal depleted and pathogen enriched, often with taxa associated with periodontal inflammation [78]. Interestingly, newer 16S rRNA studies have found a lower prevalence of conventional pathobionts, highlighting the importance of other non-conventional species in the peri-implant disease pathogenesis process [79,80,81]. Furthermore, the presence of opportunistic microorganisms such as *P. aeruginosa*, *S. aureus*, and *C. albicans* has been reported, and these may also contribute to peri-implant bone loss [82,83]. While peri-implant health is associated with a symbiotic equilibrium, factors that promote biofilm growth, and possibly different clustering patterns of biofilm, will lead to inflammation that commonly precedes loss of peri-implant bone [84].

### Factors Predisposing to Biofilm Accumulation

Appropriate prosthetic constructions that do not impede oral hygiene measures and allow for the establishment of proper and sustainable dimensions and morphology of the peri-implant tissue [85] are key to reducing the risk of peri-implantitis [86]. Other prosthetic elements associated with increased risk include the restorative contour and emergence angle [87,88], mucosal emergence angle [89], sulcus depth [90], and the position of the restoration margin relative to the crestal bone [91]. Studies also indicate that deeper crown cementation margins and concave abutments are associated with a greater quantity of excess cement [92,93,94], and this could also increase the risk for peri-implant diseases [95]. Prosthetic factors influencing biofilm control may be implant site specific, leading to different microbiological stable states in the same individual. Unfavourable prosthetic design in restorations supported by multiple implants might influence all involved implants, partly explaining the frequently observed clustering of peri-implantitis [96], but also the association often observed between peri-implantitis and technical complications [19].

Ill-positioned implants influence the path of insertion of a prosthesis and could result in poorer prosthetic contours [97], which can also increase the occurrence of peri-implantitis [98]. The latest studies also demonstrated that anatomical/morphological features of the peri-implant tissue at the respective site such as the presence of keratinised mucosa also have an impact on peri-implant health [99,100]. Indeed, soft tissue augmentation promotes peri-implant health over disease [101,102], and this could be beneficial in both the prevention and treatment of peri-implantitis [103].

## 6. Wound Healing in the Presence of a Foreign Body Results in Subclinical Inflammation

When foreign materials are introduced into the body, they elicit a response from immune cells, which attempt to envelop and degrade them. This inflammatory and fibrotic process is known as a foreign body reaction and has also been described in the medical literature, for example in nerve neuroprosthetics [104]. Albrektsson and colleagues have described ‘osseointegration’ as a foreign body equilibrium in bone, characterised by mild chronic inflammation and physical separation of the implant surface from organic structures [105]. This interface is thought to exist in a delicate balance, where various implant, clinician, and patient-related factors interact with each other.

The process of implant surgery results in trauma to surrounding tissues and triggers an acute inflammatory response, which may be considered part of physiological wound healing. The presence of titanium also activates the immune system, including macrophages and the complement system, while bone resorption is downregulated [106]. Over a period of a few weeks to months, the transition from acute inflammation to mild chronic inflammation and the formation of foreign body giant cells signals the end of the wound-healing response [107]. The presence of multinucleated giant cells may be a part of the normal osseointegration process [108]. Chappuis et al. also reported the occurrence of multinucleated giant cells on implant surfaces (titanium or zirconia) as a common finding and one that did not appear to impede peri-implant bone formation [109]. However, other authors have found that peri-implant mucosa settles in a state of ‘subclinical chronic inflammation’ compared to periodontal tissues [110]. This may explain why bleeding on probing, while uncommon in a healthy periodontium, is found around most healthy peri-implant tissues [111].

The role of titanium also presents as another confounding variable in the pathogenesis of peri-implantitis, as it may also influence the inflammatory response around the peri-implant site. This may be due to factors such as a foreign body reaction, implant surface corrosion leading to the release of titanium particles, as well as epigenetic changes. Although regarded as being biocompatible, commercial grade titanium is not fully inert. Titanium can act as an abiotic stimulus to macrophages and activate the release of the NLRP3 inflammasome complex assembly [112]. Inflammation also impairs osteoblast attachment and function, and a pro-osteoclastic phenotype has been described on infected implant surfaces [113]. However, implants may eventually integrate with surrounding tissue and return to a state of tissue balance, suggesting that a foreign body response may not be responsible for the incidence of peri-implantitis [114].

The tribocorrosive behaviour of titanium has a synergistic effect with lipopolysaccharide-primed macrophages, which results in a proinflammatory reaction [115,116]. The release of degradation products from orthopaedic biomaterials is well documented. All metallic surfaces undergo electrochemical corrosion and wear, in a process known as bio-tribocorrosion [117]. This process is exacerbated in the oral environment where dental implants are additionally subjected to pH, temperature, and saliva contamination [118]. The release of particles is unavoidable when a prosthesis is implanted and may induce inflammatory reactions which culminate in a foreign body granulation tissue response at the interface between the bone and implant [119]. The biological effects of titanium products include cell cytotoxicity, stimulation of osteoclast differentiation, stimulation of macrophages, and damage to oral epithelial homeostasis [120]. Titanium products may also function as abiotic immune activation signals, activating pro-inflammatory responses and inducing osteoclastogenesis [121]. The direct (immune modulation) and indirect (microbiome perturbation) effects are of concern, as there is potential for systemic dissemination [122]. The magnitude of detrimental changes on the peri-implant tissues is determined by the quantity, size, and chemical composition of the degradation products [123,124].

Titanium dissolution products have also been associated with epigenetic changes in peri-implant tissues and disturbances to the peri-implant microbiome [125,126]. Recent evidence has also shed light on the host–microbiome interactions in peri-implantitis lesions. Disruptions to the microbial balance around diseased implants led to a breakdown in host-bacterial interactions found in health, leading to the description of peri-implantitis as a chronic non-resolving wound [127]. The finding of titanium products in the peri-implant tissues is not uncommon, although the amount of particles present appears to be correlated with peri-implantitis sites [128,129,130]. While this association could suggest a contributory role of titanium products to the inflammatory process, other authors suggest this correlation is more likely to be a consequence rather than the trigger of disease [131]. Nevertheless, titanium products may be released during implant bed preparation, implant insertion, and bio-tribocorrosion over the lifetime of the implant [132]. The surface roughness of the implant and the overall surface area of the bone–implant contact could be related to the amount of titanium products released [133,134]. More research on this is warranted.

## 7. Genetic Risk Factors Could Predispose Patients to Peri-Implantitis

It has been reported that genetic polymorphisms could be associated with increased peri-implant tissue destruction, thus predisposing to peri-implantitis [135,136,137]. Potential genetic risk factors include genetic polymorphisms in interleukins, tumour necrosis factor (TNF)-α, matrix metalloproteinases, and growth factors involved in bone metabolism [138]. Genetic polymorphisms in these cytokines may favour inflammatory and osteolytic processes and may be associated with an increased risk of peri-implantitis and bone loss [139]. Furthermore, the loss of bone in peri-implantitis lesions is associated with the upregulation of inflammatory mediators and matrix metalloproteinases, together with an increase in fibro-osteoblastic cells which generate a tissue that is more fibrous with less osteogenic potential [140]. Other studies have also found that an increase in cytokine levels associated with relevant genetic polymorphisms may lead to increased peri-implantitis susceptibility and eventual implant failure [141,142,143]. Cigarette smoking and epigenetic factors could also have an additive effect with other genetic polymorphisms, and their subsequent role in modulating the inflammatory response to bacterial challenge may also result in a higher risk of peri-implant bone loss [139,144,145]. However, there remains a small number of studies evaluating the association between genetic polymorphisms and peri-implant disease, meaning that further investigation on this link is required before making the recommendation for routine genetic testing [146].

## 8. Macrophage Polarisation May Be the ‘Switch’ That Activates Osteolysis and Drives Pathogenesis in the Bone Dysregulation-Inflammation-Biofilm Model

Macrophages are immune cells that have a prominent role in bone homeostasis and the osseointegration of dental implants [147]. They have been found to rapidly accumulate on implant surfaces prior to bone formation, and their presence positively mediates new bone formation and mineralisation of bone [148,149]. Plasticity is a feature of macrophages, which may display an M1 (pro-inflammatory, role in host defense) or M2 (anti-inflammatory, role in tissue remodelling) phenotype [150]. The M1/M2 axis describes the opposing activities of macrophages, with M1 impairing cell proliferation and promoting tissue damage, and M2 promoting cell proliferation and tissue repair [151]. As a key part of the innate immune system, M1 and M2 macrophages are functionally polarised in response to bacteria [152], inflammatory diseases such as obesity and insulin resistance [150], and autoimmune diseases [153]. In osteoimmunology, osteolysis occurs to remove the cause of inflammation and results in bone loss and suppression of bone formation [154]. Therefore, macrophage polarisation could be an important part of the pathogenesis of peri-implantitis.

In the context of peri-implantitis, the continuous release of titanium bio-tribocorrosion products may result in a tipping point that subsequently induces M1 macrophage polarisation, leading to osteolysis [155,156,157]. In a clinical study, titanium oxide stimulation resulted in macrophage activation and the release of pro-inflammatory cytokines exceeding physiological limits in 3 out of 10 patients [158]. Notably, examples of pro-inflammatory cytokines released such as TNF-α, IL-6, and IL-11 can induce osteoclast formation independent of the RANKL coupling mechanism [159]. Other clinical studies have also reported significantly higher M1 polarisation at peri-implantitis sites [160], and a possible association with lesion severity [161]. On the other hand, intervention studies have found that neutralising the M1 macrophage polarisation response by using antibodies to IL-1β, IL-6, or TNF-α, or the introduction of resolving macrophages enabled attenuation of inflammatory osteolysis [155,162]. The immune response to implant-derived wear particles also provides a biological basis for aseptic implant failure [163].

## 9. Clinical Implications and Further Research

**Figure 3:**
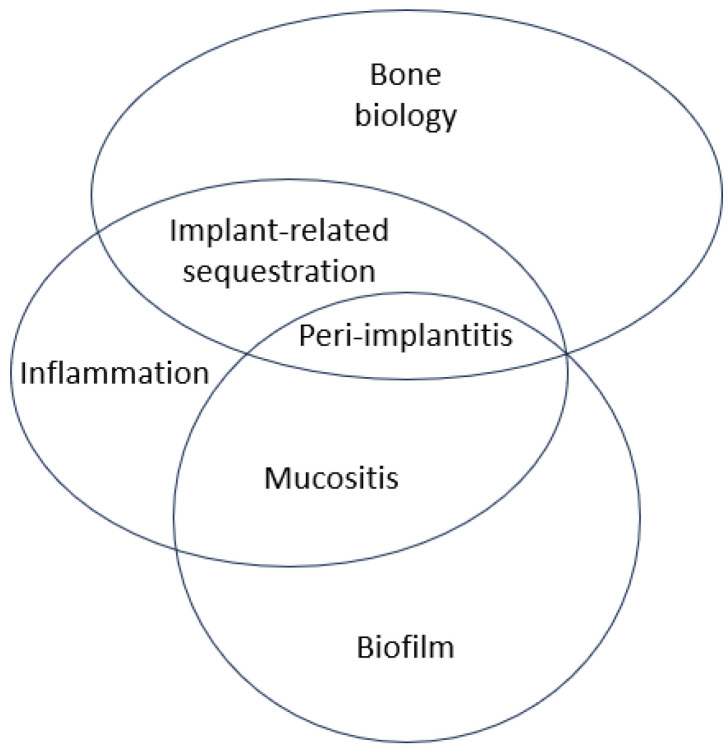
Hypergraph depicting the relationship between the core components of BIND and known peri-implant conditions.

The stages of the BIND hypothesis may be summarised as follows: (I) physiological homeostasis (peri-implant tissue health); (II) pathological homeostasis (multi-stable states and hysteresis); (III) pathological exacerbation and tipping point; (IV) pathological dysregulation (peri-implantitis). Current evidence suggests that immune-inflammatory induced differentiation of osteoclasts can explain the uncoupled and accelerated bone resorption in peri-implantitis lesions. The framework introduced in this paper explains the transition from health to disease as a staged and incremental process, where multiple factors can contribute to steps toward a tipping point culminating in clinical disease. The core components of BIND can be used to map contributory factors to known peri-implant conditions (Figure 3). An improved understanding of disease pathogenesis also allows for intervention on multiple levels and a personalised treatment approach. Nevertheless, this manuscript has some limitations. While some of the factors mentioned have been documented in the literature, their relative contributions to multi-stable states and hysteresis tipping points are unquantified and may not be equal. Also, some of these factors are interrelated or even overlapping, and it is difficult to identify their exact mechanisms.

The loss of an implant from advanced bone loss due to peri-implantitis is a terminal event that can be caused by a dysregulated immune response. This may occur regardless of the implant material (titanium or zirconia) but could possibly be exacerbated by titanium dissolution products. Therefore, it is necessary to clarify the biological effect of titanium products to better understand their potential impact on peri-implant inflammation, tissue repair, and treatment interventions. Further investigations are needed to identify synergistic factors that lead to the breakdown of osseointegration, and to identify other methods of decontamination that can effectively remove biofilms while maintaining the integrity of the implant surface.

A better understanding of the disease pathogenesis process will also facilitate the development of novel early detection methods of peri-implant bone loss, such as the use of biomarkers to identify changes in macrophage polarisation and activation status. CD68, CD80, iNOS, and CD206 are currently used to analyse M1/M2-mediated destruction, and possible therapeutics modulating this axis may promote disease resolution and enhance tissue repair. TiO2 stimulation tests may also be a useful tool in estimating a hyperresponsive macrophage phenotype, which is positively associated with peri-implantitis. MicroRNAs (miRNAs) have also emerged as a promising epigenetic biomarker for bone diseases, in contrast to bone turnover markers which evaluate bone cell metabolic activity and have limitations as diagnostic or prognostic markers [164]. MiRNAs are short sequences of non-coding RNA that have a significant role in regulating gene expression, and their role in regulating bone metabolism on a genetic level has been previously described [165]. They are convenient to sample since circulating miRNAs are easily detectable in biofluids such as serum, and more importantly, enable the assessment of the epigenetic environment since they act as modulators rather than effectors of biological function [166]. This suggests that they could be detectors of bone dysregulation on a molecular level before clinical signs become apparent. Indeed, in clinical studies of osteoporosis patients, circulating miRNAs showed superior performance over traditional bone turnover markers in distinguishing osteoporosis patients from healthy and osteopaenic patients [167], and could be predictive of vertebral fractures in low-bone-mineral-density patients [168].

## 10. Conclusions

The BIND hypothesis is proposed where peri-implantitis pathogenesis involves multiple interactions between microbial biofilm, bone biology, and inflammation mechanisms. Factors affecting these components contribute to different microbiota profiles within a multi-stable state system in the local implant microenvironment. The transition from health to disease is a staged and incremental process, before reaching a tipping point where disease is manifested clinically. The components of BIND may also be used to map factors that correlate clinically with known peri-implant conditions. Identifying individual- and site-specific factors for the breakdown of osseointegration will lead to a personalised approach for peri-implantitis risk assessment and therapy.

## Figures and Tables

**Table 1:** Description of terms.

Peri-implant tissue health	The absence of clinical signs of inflammation, and can include successfully treated peri-implant disease with variable levels of bone support
Peri-implant disease	Collective term for peri-implant mucositis and peri-implantitis
Risk factor	An environmental, behavioural, or biological factor that if present directly increases the probability of a disease occurring and, if absent or removed, reduces that probability, based on epidemiologic evidence, usually in prospective cohort studies
Risk indicator	Putative risk factors tested until their significance as true risk factors are proven or rejected
Microbial dysbiosis	The change in abundance of species that were already present at baseline or from health, peri-implant mucositis to peri-implantitis
Microbiota	Describes the situation of a mixed microbial population
Multi-stable state system	The dynamic property of a system that exhibits multiple mutually exclusive stable states. Microbial communities for each state exist with different species abundance profiles
Hysteresis	The innate resistance in a system before reaching a different state
Non-susceptible individuals	Individuals with various exposure to the presented factors, who are less likely to develop clinical disease due to a well-regulated homeostatic immune response
Susceptible individuals	Individuals with various exposure to the presented factors, who are more likely to develop early (mucositis) or late (peri-implantitis) disease due to a dysregulated homeostatic immune response
Bio-tribocorrosion	The combination of tribology (friction and wear), and corrosion with the biological environment
